# Editorial: Accelerating antibiotic development from natural products: tackling antimicrobial resistance (AMR)

**DOI:** 10.3389/frabi.2026.1898318

**Published:** 2026-06-29

**Authors:** Sanjib Bhakta, Cynthia Amaning Danquah, RuAngelie Edrada-Ebel, Simon Gibbons

**Affiliations:** 1 School of Natural Sciences, Institute of Structural and Molecular Biology, Birkbeck, University of London, London, United Kingdom; 2 Department of Pharmacology, Faculty of Pharmacy and Pharmaceutical Sciences, Kwame Nkrumah University of Science and Technology (KNUST), Kumasi, Ghana; 3 Strathclyde Institute of Pharmacy and Biomedical Sciences, University of Strathclyde, Glasgow, United Kingdom; 4 Centre for Natural Products Drug Discovery (CNPDD), Department of Pharmacy and Biomolecular Sciences, Liverpool John Moores University, Liverpool, United Kingdom

**Keywords:** adjunctive therapies, antibiotic discovery, antimicrobial resistance (AMR), biofilm, drug efflux, natural products

## Abstract

**Figure:**
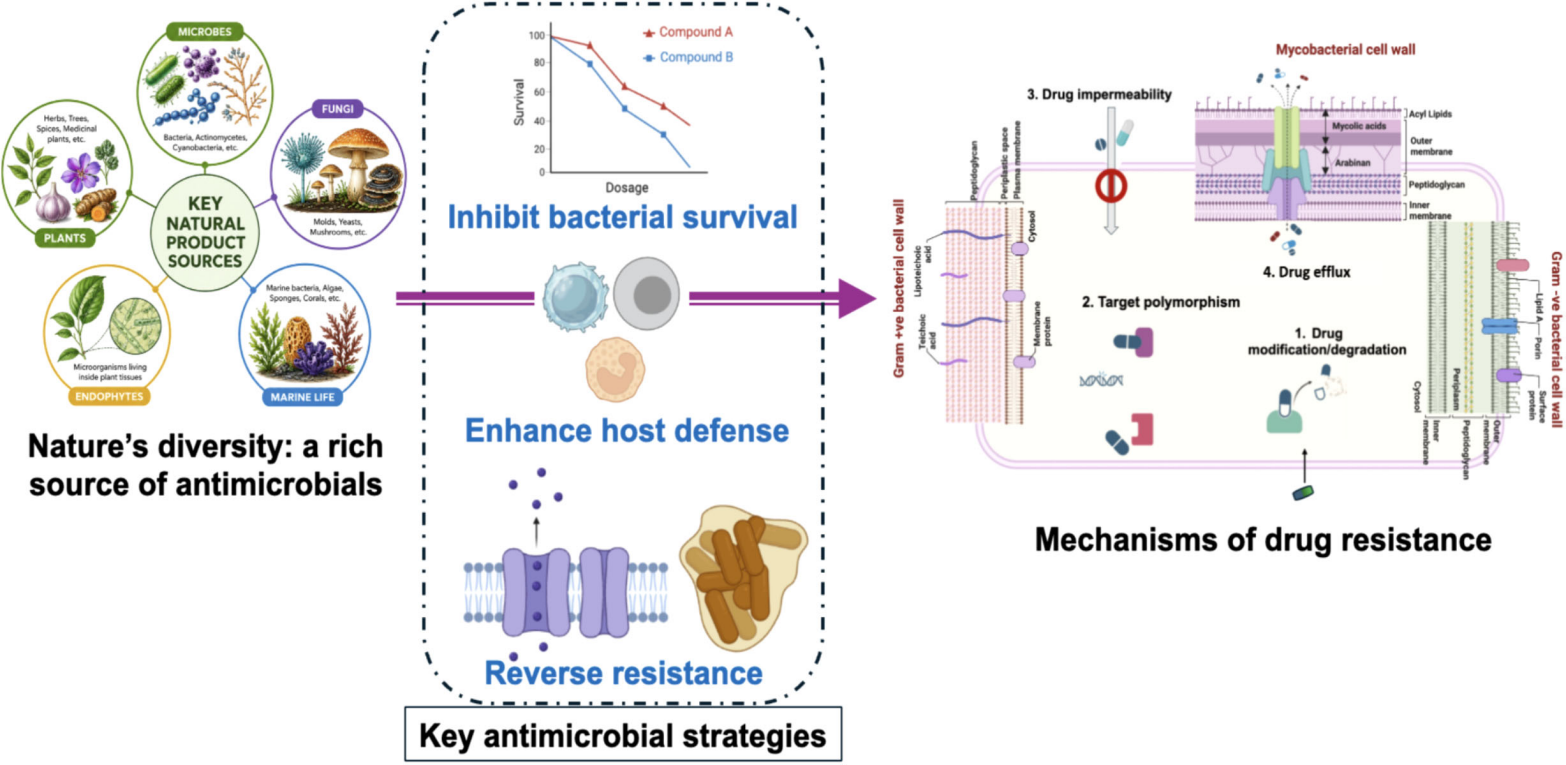
Accelerating antibiotic development from natural products. Natural products with antimicrobial activity can act through multiple mechanisms to overcome antibiotic resistance. These include directly inhibiting bacterial growth, reversing resistance by blocking efflux pumps and disrupting biofilms, and strengthening host defenses. Together, these strategies help restore antibiotic effectiveness, overcome resistance, and support more sustainable approaches to treating infections. Image created by Dr Ankita Nag in BioRender. (https://BioRender.com/6c0p6qp), licensed under CC BY 4.0.

Accelerating antibiotic development from natural products. Natural products with antimicrobial activity can act through multiple mechanisms to overcome antibiotic resistance. These include directly inhibiting bacterial growth, reversing resistance by blocking efflux pumps and disrupting biofilms, and strengthening host defenses. Together, these strategies help restore antibiotic effectiveness, overcome resistance, and support more sustainable approaches to treating infections. Image created by Dr Ankita Nag in BioRender. (https://BioRender.com/6c0p6qp), licensed under CC BY 4.0.

Antimicrobial resistance (AMR) continues to threaten global public health, undermining decades of progress in infectious disease treatment and modern medicine. Current projections suggest that, if left unchecked, drug-resistant infections may claim more lives annually than cancer by 2050. Against this backdrop, the urgent need for innovative anti-infective strategies has renewed scientific interest in natural products as a rich and evolutionarily refined reservoir of antimicrobial agents. Natural compounds derived from plants, microbes, marine organisms and associated symbiotic microbiota possess unparalleled chemical diversity, structural complexity and biological specificity that often remain difficult to replicate synthetically. This Research Topic, *Accelerating Antibiotic Development from Natural Products: Tackling Antimicrobial Resistance (AMR)*, brings together a diverse collection of studies that collectively highlight the contemporary advances, challenges and translational opportunities in natural-product-driven antimicrobial discovery.

Historically, nature has served as the creative foundation of antibiotic discovery. Many clinically important antibiotics, including β-lactams, aminoglycosides and tetracyclines, originated from natural sources. However, the rediscovery of known compounds, limited commercial incentives and increasing resistance have contributed to the slowdown in antibiotic innovation. Recent advances in genomics, metabolomics, artificial intelligence/machine learning (AI/ML), molecular docking and dynamic simulation, synthetic biology and high-throughput phenotypic screening are now revitalizing the field by enabling the identification of previously inaccessible or cryptic bioactive molecules.

The studies assembled in this Research Topic exemplify this resurgence and demonstrate the broad spectrum of approaches being employed to address AMR through natural products and biologically inspired therapeutics. Several contributions focus on phytochemicals and plant-derived compounds with direct antimicrobial or anti-virulence properties. Alhodieb et al. explored the bioactive constituents of *Carica papaya* leaves and identified phytol as a promising antibacterial agent against antibiotic-resistant pathogens. Their work provides mechanistic insight into membrane disruption and oxidative stress induction, further reinforcing the therapeutic potential of medicinal plants as accessible sources of antimicrobial leads.

Similarly, Salih et al. investigated phenolic extracts from sorghum and demonstrated significant antimicrobial activity against liver abscess-associated bacterial pathogens. Their findings support the growing evidence that agricultural crops and dietary plants may contain underexplored antimicrobial phytochemicals with translational potential in both human and veterinary medicine.

Beyond direct bactericidal activity, several studies emphasized alternative strategies aimed at attenuating bacterial pathogenicity and resistance mechanisms. Ying et al. demonstrated that cyano-phycocyanin acts as a quorum-sensing inhibitor capable of suppressing *Pseudomonas aeruginosa* virulence. Anti-virulence approaches are increasingly attractive because they exert reduced selective pressure for resistance compared with conventional antibiotics while disarming pathogens by targeting communication systems, biofilm formation and virulence regulation.

The challenge of biofilm-associated resistance was addressed by Wang et al., who investigated biofilm-mediated resistance to berberine in *Escherichia coli*. Their work highlights the complex adaptive responses associated with natural-product exposure and highlights the importance of understanding resistance development even against compounds traditionally perceived as less resistance-prone. Such studies are essential for translating natural products into clinically viable therapeutics while anticipating evolutionary responses by microbial pathogens.

Combination therapy and synergistic antimicrobial strategies also emerge as major themes within this Research Topic. Shen et al. reported drug-drug interactions between mupirocin and protocatechuic acid ethyl ester against mupirocin-resistant methicillin-resistant *Staphylococcus aureus* (MRSA). The study elegantly demonstrates how natural-product-derived adjuvants may restore antibiotic efficacy against resistant pathogens, thereby extending the lifespan of existing antimicrobial agents. Combination therapies that target multiple bacterial pathways simultaneously may also reduce the probability of resistance emergence.

Likewise, Mundhe et al. integrated *in silico* molecular docking with experimental validation to identify isosilybin A as an antifungal adjuvant targeting squalene epoxidase (ERG1) in azole-resistant *Candida* species and emerging yeasts. This contribution reflects the increasingly important role of computational biology and AI-assisted screening in accelerating natural-product-based drug discovery pipelines. Integrating *in silico* predictions with experimental microbiology can substantially reduce time and cost barriers associated with traditional antimicrobial discovery.

Another important dimension represented in this Research Topic is the growing interest in antimicrobial peptides (AMPs) as next-generation therapeutics. Tao et al. demonstrated the potent anti-*Pseudomonas aeruginosa* activity of AMP-17, including efficacy in wound infection models. The ability of AMPs to disrupt bacterial membranes, modulate immune responses and reduce resistance development makes them highly attractive candidates for future antimicrobial therapy. Complementing this, Das et al. reviewed the emerging role of AMPs in sustainable plant disease management, highlighting their relevance beyond human medicine and reinforcing the interconnected One Health dimensions of AMR.

Panigrahy et al. and Shen et al. further broadened the conceptual scope of the topic through their mini-review discussing the expanding horizons of microbial natural products beyond traditional antibiotics. Their article highlights how microbial metabolites may function not only as antimicrobials but also as signaling molecules, immunomodulators and anti-biofilm agents. This evolving perspective aligns with contemporary understanding that microbial secondary metabolites participate in complex ecological interactions extending far beyond simple competitive inhibition.

Another notable review by Sahoo et al. examined the multifaceted biological applications of EDTA in the context of microbial modulation and natural-product research. By discussing EDTA’s effects on membrane permeability, biofilm disruption and synergistic enhancement of antimicrobial activity, the authors provide important mechanistic insights relevant to combination therapies and antimicrobial sensitization strategies.

Traditional medicine and ethnopharmacology also feature prominently within this Topic. Xu et al. investigated the efficacy of *Radix Paeoniae alba* against pneumococcal toxicity through targeting pneumolysin. Their findings illustrate how bioactive compounds derived from traditional medicinal systems may provide novel anti-virulence strategies that complement or enhance conventional antimicrobial therapy.

Collectively, the scholarly contributions within this Research Topic demonstrate several important emerging trends in antimicrobial discovery. First, there is a clear transition from solely pursuing bactericidal agents toward targeting virulence, quorum sensing, biofilm formation and host-pathogen interactions. Second, interdisciplinary integration of microbiology, chemistry, computational biology and systems pharmacology is becoming essential for accelerating discovery pipelines. Third, the convergence of natural-product chemistry with AI-driven screening, molecular docking and synthetic biology offers unprecedented opportunities to identify structurally novel antimicrobial scaffolds and optimize their therapeutic potential.

Importantly, this Research Topic also highlights continuing challenges. Many natural products face translational barriers including limited bioavailability, toxicity concerns, difficulties in large-scale production and insufficient pharmacokinetic characterization. Marine-derived compounds and symbiotic microbial metabolites remain particularly underexplored despite their immense biosynthetic potential. Future success will require stronger integration between biodiversity exploration, sustainable bioprospecting, medicinal chemistry optimization and translational clinical research.

The global AMR crisis cannot be addressed through a single therapeutic paradigm. Instead, it demands diversified and innovative approaches encompassing novel antibiotics, antimicrobial adjuvants, anti-virulence therapies, host-directed interventions and precision antimicrobial strategies. The studies presented in this Research Topic collectively reinforce the enduring relevance of natural products as one of the most promising frontiers for antimicrobial innovation.

We sincerely thank all authors, reviewers and editorial staff whose contributions made this Research Topic possible. We hope this Research Topic will stimulate further interdisciplinary collaboration, inspire innovative antimicrobial discovery efforts and contribute meaningfully toward global strategies aimed at combating AMR and safeguarding the future of anti-infective therapy.

